# High‐throughput methods for measuring protein extractability in sugar beet ( *Beta vulgaris* L.) leaves

**DOI:** 10.1002/jsfa.70074

**Published:** 2025-07-21

**Authors:** Hugo Rijken, Hannah F Oertel, Christian B Hirschmann, Alain Tossens, Dieter Hackenberg, Juan Vegas, Marieke E Bruins, Luisa M Trindade

**Affiliations:** ^1^ Plant Breeding Wageningen University & Research Wageningen Netherlands; ^2^ KWS SAAT SE & Co. KGaA Einbeck Germany; ^3^ United Beet Seeds BV Tienen Belgium; ^4^ Wageningen Food & Biobased Research Wageningen University & Research Wageningen Netherlands

**Keywords:** phenotyping, nitrogen conversion factor, TCA precipitation, side stream valorization, alternative proteins

## Abstract

**BACKGROUND:**

Most components from the roots of sugar beet (*Beta vulgaris* ssp. *vulgaris*) are valorized by industry. However, the leaves are currently left on the field, even though they contain large amounts of protein. To support leaf protein valorization, high‐throughput methods and phenotyping tools were developed to facilitate the selection of beet varieties with superior protein production. This study presents high‐throughput methods to measure total protein content in leaves and to determine the amount of soluble protein extracted through pressing, enabling the calculation of leaf protein extractability.

**RESULTS:**

The influence of harvested leaf sample size and within‐plant leaf selection on protein measurements is demonstrated. Representative samples of a plot entailed collection of the middle leaves from a minimum of 25 plants. To determine total leaf protein, a near‐infrared‐based model was developed, exhibiting excellent predictive performance. Protein measurements on leaf protein extracts, based on total nitrogen, were found to correlate strongly with RuBisCO quantification obtained through size‐exclusion chromatography. Non‐protein nitrogenous compounds were measured to assess their impact on protein estimates derived from total nitrogen measurements. A strong correlation between total nitrogen and proteinogenic nitrogen was observed, confirming total nitrogen as a reliable indicator of true protein content in sugar beet leaves.

**CONCLUSION:**

This study provided high‐throughput methods for assessing leaf protein content and extractability in sugar beet. The strong correlation between total nitrogen and true protein confirms their reliability for protein quantification. These findings aid efficient screening of sugar beet germplasm for improved leaf protein yield, contributing to sustainable leaf valorization. © 2025 The Author(s). *Journal of the Science of Food and Agriculture* published by John Wiley & Sons Ltd on behalf of Society of Chemical Industry.

## INTRODUCTION

On 1.4 million hectares in Europe, sugar beet (*Beta vulgaris* ssp. *vulgaris*) is grown for the production of tap roots from which sugar is extracted.^1^ Besides sugar, factories processing sugar beet are able to utilize other components of the beet root, for example beet pulp and tips for the production of green gas or bioethanol.^2^ The sugar extraction process also yields other valuable byproducts such as natural dyes (betaine and vulgaxanthin) and calcium‐rich fertilizers.^3^ This can be seen as a good example of maximizing the value of the harvested product – the beet root. However, about 20–34% of the wet weight of the sugar beet plant are leaves. It is estimated that nearly 29 t ha^−1^ of sugar beet leaves are available every year,^4^ but they are currently not utilized and discarded onto the field, even though there is potential to valorize them as a source polyphenols, fibers, chlorophylls and proteins in a biorefinery.^5^ About 22.8% of the dry matter of sugar beet leaves consists of crude protein.^6^, ^7^, ^8^ A considerable portion of this is the photosynthesis enzyme ribulose‐1,5‐biphosphate carboxylase/oxygenase (RuBisCO). RuBisCO extracted from leaves is highly digestible, and contains all of the essential amino acids.^7^, ^9^ Furthermore, RuBisCO has remarkable functionalities, including good gelling, foaming and emulsifying properties. RuBisCO protein isolates showed more stable foams, and self‐supporting gels were obtained at lower concentrations compared to whey and soy protein.^10^ RuBisCO also showed similar gelling properties to egg white protein, making it a potential replacer for this animal‐derived protein.^11^, ^12^ Plant‐based proteins as food ingredients are increasingly in demand, due to the growing market of plant‐based food alternatives.^13^

A major hurdle to valorizing leaf protein is that protein extraction yield is generally low, due to a low extractability of 6% for sugar beet.^14^ To overcome low protein extraction yield, researchers are aiming to optimize extraction processes, and to understand which factors are hindering protein extractability.^15^, ^16^ One of these factors is thought to be the cell wall, which acts as a physical barrier and has to be severed to release the soluble proteins within the cell.^15^ Enzyme‐aided cell wall disintegration has been shown to increase protein extractability from sugar beet leaves.^17^ Besides the cell wall, another factor could be the activity of proteolytic enzymes which break down the proteins into smaller peptides, and therefore increase the ratio of non‐protein‐nitrogen (NPN) to protein.^18^, ^19^ In addition to modifying the extraction process, protein extraction yield may be enhanced through the development of new sugar beet varieties that are better suited for leaf protein extraction. Selective breeding could create new varieties that contain more leaf protein, or perhaps change the cell wall characteristics or protease activity, leading to improved protein extractability. Leaf protein extractability has not yet been studied in the context of large‐scale breeding trials for sugar beet. These trials require a large number of genotypes to ensure sufficient germplasm diversity, along with replications and multi‐environment testing, leading to a rapid increase in sample numbers. This presents a challenge, highlighting the need for effective approaches to measure protein extractability in breeding trials.

Accurate phenotyping of protein extractability starts with sampling the leaves in a way that is representative for the plot. Research shows that there can be large differences in protein recovery within leaves from the same plant,^15^, ^20^ and there is a trend of decreasing protein extractability with leaf age. Furthermore, guidelines for variety trials in sugar beet require 80–100 beets for a representative estimation of root yield,^21^ indicating that sugar beet plots can be heterogeneous. Currently, there is no information on the variation between leaves from the same sugar beet plant for leaf protein extractability. Likewise, the necessary number of plants to be sampled to obtain a representative sample is unknown. In studies of protein extraction from sugar beet leaves, the leaves are often sampled without the petiole, but the number of plants or which leaves were sampled is often not mentioned.

Besides optimal leaf sampling procedures, how to accurately measure leaf protein is also a point of discussion. Leaf protein content is often obtained by measuring the total nitrogen content and converting to protein with a nitrogen‐to‐protein conversion factor. In sugar beet leaves, this factor has been reported to vary from 4.32 to 4.95, with younger plants having a higher conversion factor.^22^ This finding indicates varying fractions of NPN in sugar beet leaves. According to Peréz‐Vila *et al*.,^23^ NPN fractions in leaves can vary widely, therefore suggesting that it is important to determine the fraction of NPN in addition to the total nitrogen content. It is unknown how much the proportion of NPN varies between different sugar beet genotypes. If there is large variation in the NPN fraction, total nitrogen becomes inadequate as a selection criterion for increased leaf protein. Therefore, the extent of variation in the NPN fraction from different sugar beet genotypes and environments has to be studied.

Besides accuracy, the large number of samples in breeding trials requires phenotyping methods with a manageable workload per sample. Leaves can be particularly challenging to handle due to their high water content, making them prone to degradation. This creates storage difficulties and imposes a time constraint on analysis following sampling.^24^ The current process of protein recovery from leaves is laborious. Firstly, it requires mechanical disruption to open the plant cells and release the cellular components. Then, the juice is separated from the pulp through filtration. The resulting juice requires further separation of the soluble and insoluble fractions by centrifugation, heating and/or acid precipitation. The protein content of the separated (soluble) fraction is then measured and compared to the protein content of the total leaves to obtain protein extractability. While the steps to obtain the protein extract are difficult to optimize, the methods to measure the protein content may be adapted to enable high‐throughput screening.

A widely used tool for high‐throughput phenotyping is near‐infrared spectroscopy (NIRs). Nitrogen‐based protein content has been shown to be highly predictable with NIRs.^25^ NIRs was used to predict the protein content of dried and milled cowpea leaves.^26^ Furthermore, NIRs was used to predict the nitrogen and protein content of sugar beet leaves with good prediction results, although the sample size was small.^27^ Beyond mere contents of samples, NIRs has also been used to determine the saccharification efficiency of biomass crops.^28^, ^29^ Furthermore, NIRs was used to predict the bioethanol yield of sugar beet pulp.^30^ These NIRs applications go beyond predicting the mere contents of samples, but extend to predicting the outcome of a process or conversion of the sample. It is therefore interesting to investigate whether NIRs can be applied to predict leaf protein extraction yield, or extractability. The use of this high‐throughput method would vastly speed up the phenotyping process.

In the study reported here, tools for measuring protein extractability in the context of large‐scale breeding trials were developed. To understand how to obtain a representative sample of leaves from a plot, two different experiments were carried out. Firstly, inner, middle and outer leaves were compared for leaf protein content, yield and extractability. Secondly, multiple samples of different plants from the same plot were taken to determine the variation among plants from the same plot. This information was used to establish optimal sampling procedures and to determine the necessary number of plants to obtain a representative sample from a plot. It was also investigated whether total nitrogen measurements are adequate as a measurement of leaf protein content. To assess this, total nitrogen measurements were compared with those of proteinogenic nitrogen and RuBisCO. Lastly, the advantages of NIRs as a phenotyping tool for measuring protein extractability were evaluated.

## METHODS

### Material: field trials

A diverse set of leaf samples was used within this study, collected from eight field trials conducted in 2022 and 2023 (Table 1). The plants grown on these fields were three‐way hybrids, part of active breeding programs of seed companies KWS SAAT SE & Co. KGaA and SESVanderHave NV. In the KWS trials, 200 hybrids were tested with two replicates per trial, while the SV trials evaluated a different set of 180 hybrids, also with two replicates. A set of 20 commercial varieties from both companies was shared across all trials. The majority of the samples were used to create NIRs prediction models (section NIRs model development). Certain subsets of samples were selected for specific analyses within this study. For example, a subset of 48 samples from the trials in 2022 was used to examine the ratio of proteinogenic nitrogen to total nitrogen (section Nitrogen fractions). In August of 2023, before the main harvest of the complete trials, 85 samples were harvested from trials T1‐23 and T5‐23 to investigate the variation between leaves within plants, and between samples from the same plot (section Sampling experiments). Another subset of 150 samples from the 2023 trials was measured for RuBisCO content (section RuBisCO). These data were used to investigate the correlation between RuBisCO and total nitrogen measurements.

**Table 1:** Sugar beet field trials and their locations

Panel	Trial	Location	Year
KWS	T1‐22	Lelystad (NL)	2022
	T2‐22	Lens (FR)	2022
	T1‐23	Lelystad (NL)	2023
	T3‐23	Lens (FR)	2023
SV	T4‐22	Lelystad (NL)	2022
	T5‐22	Roosendaal (NL)	2022
	T6‐23	Lelystad (NL)	2023
	T5‐23	Roosendaal (NL)	2023

### Sampling

The samples were harvested at the end of the growing season. Only plants from the middle row of a plot were collected. In 2022, all leaves from 10 plants were collected. In 2023, 3–4 leaves were sampled from each plant in the middle row. Leaves were collected without the petiole and put in 50 cm × 80 cm monofilament net bags to create one bulk sample per plot.

#### Sample processing

The bulk leaf sample was mixed and divided into two subsamples. Subsample A was collected in monofilament net bags and used to measure the dry matter and total protein content. Subsample B was used to determine the percentage of juice obtained through pressing and the yield of extracted protein. Subsample B was collected in an airtight and light‐proof aluminium zipbag (30 cm × 50 cm, 9 L). To minimize potential errors induced through degradation of the leaves, the subsamples were temporarily stored at 4 °C in a refrigerated truck while on location.

After sample collection, subsample A was dried at 50 °C for 48 h. The dried leaves were then weighed, milled to 1 mm (Peppink 200 AN, The Netherlands) and stored in airtight containers. Using the fresh and dry weight, the dry matter content of subsample A was calculated. The total protein content of subsample A was obtained using a NIRs‐based prediction model (section NIRs model development).

Subsample B was stored at 4 °C until weighing and pressing of the leaves using an Angel Juicer 7500 (Angel Juicer Co. Ltd, South Korea). The juice was collected and weighed to determine the percentage of juice pressed from the leaves. A 2 mL subsample of the juice was taken and centrifuged at 20 000 × *g* for 45 min to precipitate any solids. The nitrogen content of the supernatant was measured using the Dumas method. This was done by transferring 500 μL into a tin capsule (20 mm × 8 mm, elemental microanalysis), drying at 60 °C for 48 h and subsequent combustion in a rapid N‐exceed nitrogen and protein analyzer (Elementar).

The dry matter (g kg^−1^ leaves) and protein content (g kg^−1^ dried leaves) were measured in subsample A. The percentage of juice (g kg^−1^ leaves) and extracted crude protein (g kg^−1^ supernatant) were measured in subsample B. All were used to calculate the protein yield and protein extractability of the original sample before splitting. Protein yield (g kg^−1^ dried leaves) was determined as the ratio of protein in the juice and the dry matter content of the leaves, as shown in Eqn (1):

(1) $$\text{Protein yield}=\frac{\text{Percentage of juice}\;\left(B\right)\times \text{protein in supernatant}\;\left(B\right)}{\mathrm{Dry}\;\text{matter content}\;\left(A\right)}$$

The protein yield was then expressed as a percentage of total protein to obtain protein extractability (g kg^−1^ protein) using Eqn (2):

(2) $$\text{Protein extractability}=\frac{\text{Protein yield}\;}{\text{Total protein}}\times 100$$

### Sampling experiments

Field trials T1‐23 and T5‐23 were used to conduct sampling experiments. The purpose was to determine the variation between leaves within a plant, and the number of plants required to obtain a plot representative sample. Sugar beet commercial varieties KWS_199 and SV_207 were used for the leaf selection experiment, while KWS_197 and SV_204 were used for the sample size experiment. These varieties were chosen based on preliminary data indicating differences in leaf protein content. Each variety was replicated once per trial and both replicate plots were sampled. Sampling of the material for both experiments was done in parallel. All samples were measured for dry matter content, total protein content, juice percentage, protein in juice supernatant, protein yield and protein extractability.

#### Leaf selection

The rosette growth of sugar beet leaves was categorized into: (1) inner leaves from the center of the rosette, (2) outer leaves and (3) middle leaves from between the two aforementioned categories. To ensure a sufficient amount of leaves per sample, a minimum of 1.5 kg of leaves was harvested. The whole plot (*ca* 90 plants) was used for the sampling of the different categories. For middle and outer leaves, one leaf per plant was taken. Since inner leaves are smaller than middle and outer leaves, multiple inner leaves per plant were taken.

#### Sample size

To determine the optimal sample size, a method similar to that of Burba and Haufe^31^ was employed. Each plot (*N* = 8 in total) was sampled repeatedly by taking all of the leaves from 9 plants in a row (9 plants were used to obtain one sample). Between 5 and 8 samples were taken per plot, depending on the amount of plants per plot.

#### Data analysis

In the sample size experiment, the variability of samples from the same plot was assessed by calculating the plot‐based mean, standard deviation and coefficient of variation (CV) for each trait. The mean CV across all plots was used to compare the variability of the traits. To determine the benefit of sampling a larger number of plants from a plot, the CV was calculated at increased sample sizes using a custom R script (S1). This script uses all unique combinations of samples from a plot, with the total amount of samples combined increasing from 1 to 8, to simulate increasing sample size. For each combination, the mean was calculated and stored in a list. These means were then used to calculate a CV per plot, at each number of samples combined. The average CV across all plots, for each trait and at each number of samples combined was calculated and plotted. A CV of 5% was taken as an acceptable threshold for variability among samples from the same plot.

### Development of phenotyping/protein methods

The validity of total nitrogen measurements for quantifying leaf protein was assessed using two approaches. First, the fraction of proteinogenic nitrogen was determined and compared to total nitrogen measurements in dried leaf material. Second, RuBisCO content was measured in leaf juice supernatant samples and compared to the total nitrogen measurements of the same samples.

#### Nitrogen fractions

To determine proteinogenic nitrogen, NPN compounds were separated from protein by trichloroacetic acid (TCA) precipitation and then subtracted from total nitrogen. For this, milled sugar beet leaf (2 g) was mixed with 20 mL of 10% (w/v) TCA–water solution. This mixture was centrifuged for 15 min at 4 °C and 4000 × *g*. To counter the TCA acidity, 25 μL of 50% NaOH–water solution was added to a tin cup, after which 500 μL of the TCA supernatant was added. The solution was then dried overnight at 60 °C. To obtain a detectable amount of nitrogen, this procedure was repeated once, coming to a total of 1000 μL of the TCA supernatant added to the tin cup. The nitrogen content contained in the tin cup was measured with a rapid N‐exceed nitrogen and protein analyzer (Elementar).

#### RuBisCO

Frozen juice samples were defrosted and 2 mL of juice was diluted to a final sample/buffer ratio of 1:4, using a buffer containing 20 mmol L^−1^ Tris and 150 mmol L^−1^ NaCl with a pH of 8,0 to stabilize the proteins. The mixture was centrifuged for 15 min at 21 000 × *g* to spin down all fibers. From this step, 50 μL was injected onto a Superdex 200 HR 10/300 GL column (GE Healthcare). RuBisCO qualification was based on retention time, and quantification was based on the area under the curve and a single‐point calibration curve using RuBisCO as the reference protein.

### Development of NIRs models

To allow for future high‐throughput analysis of leaf protein, NIRs‐based prediction models were developed for total protein content, protein yield, RuBisCO yield, protein extractability, RuBisCO extractability, protein in juice and RuBisCO in juice. NIR spectral absorbance (400–2500 nm) of dried and milled leaves (subsample A) was recorded using a FOSS NIRs analyzer (FOSS DS2500). Spectral absorbance of juice samples (subsample B) was measured using the same machine, but with the DS2500 Slurry cup as a sample cup. To calibrate the prediction models, different subsets of samples were used. For total protein content, a set of 184 samples from 2022 was chosen based on the Mahalanobis distance. An additional 65 samples from the sample size experiment were added, resulting in a calibration set of 251 samples. The total nitrogen content of the samples was measured in duplicates of 100 mg in a rapid N‐exceed nitrogen and protein analyzer (Elementar) and corrected for dry matter content. This value was then multiplied by 6.25 to obtain crude protein values.

For the prediction of protein yield, RuBisCO yield, protein extractability, RuBisCO extractability, protein in juice and RuBisCO in juice, a calibration set of 116 samples from the 2023 trials was used. Protein yield and protein extractability were obtained using Eqns (1) and (2). RuBisCO yield and RuBisCO extractability were obtained using the same equations, except the protein in supernatant parameter in Eqn (1) was substituted for RuBisCO content as measured via size‐exclusion chromatography–high‐performance liquid chromatography (section RuBisCO).

Modified partial least squares regression was applied on the calibration set to build a prediction model for protein content across all of the samples. The performance of the model was tested on an external validation set (random subset of samples from 2023 trials) to obtain the coefficient of determination (*R* ^2^), standard deviation, standard error and ratio of performance to deviation (RPD).

## RESULTS

### Sampling experiments

#### Middle leaves yield more protein

**Figure 1:**
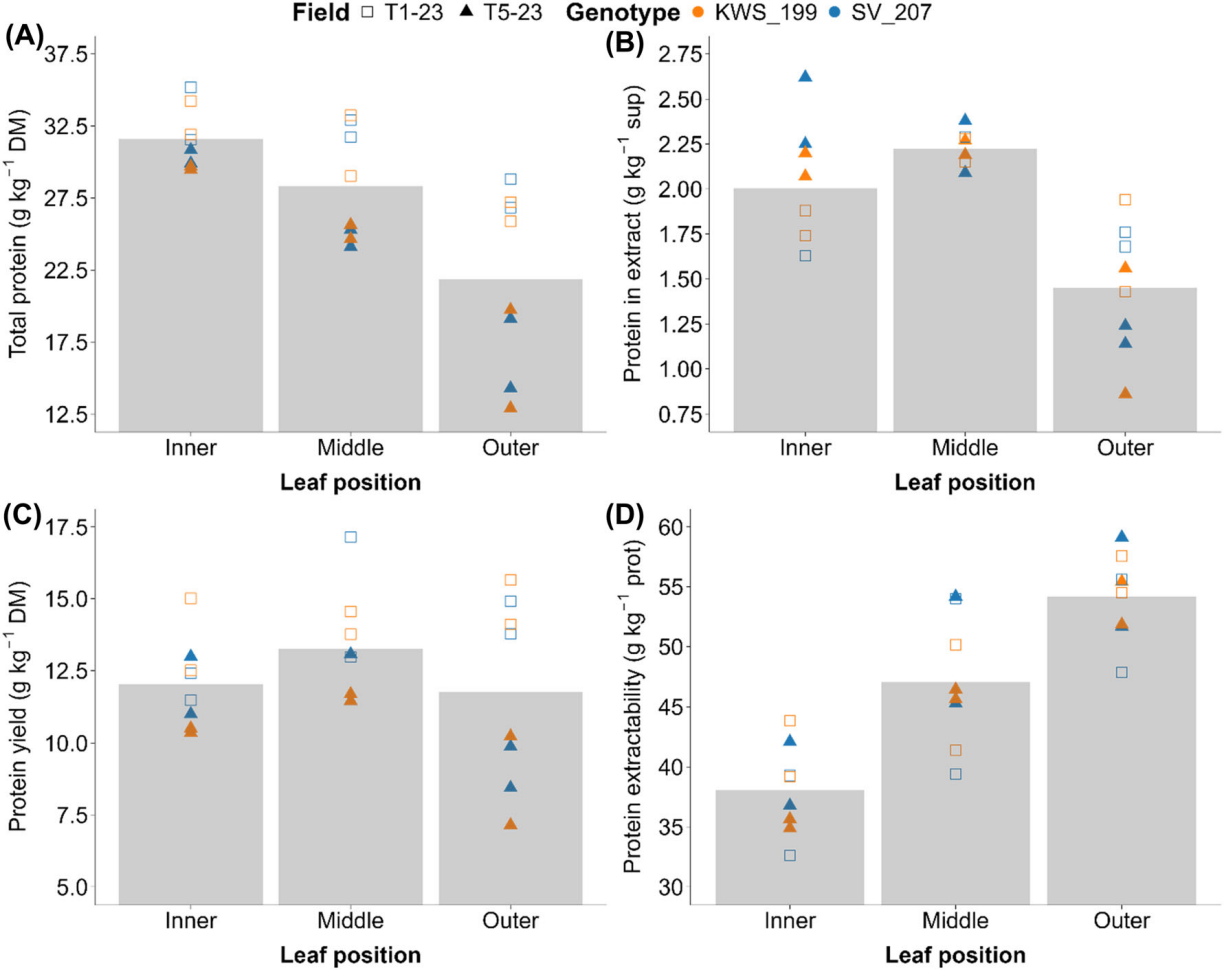
Differences between inner, middle and outer leaves for leaf protein‐related measurements. (A) Total protein (g kg^−1^ DM). (B) Protein in extract (g kg^−1^ supernatant). (C) Protein yield (g kg^−1^ DM) as calculated with Eqn (1). (D) Protein extractability (g kg^−1^ protein) as calculated with Eqn (2). Trials are indicated by the shape of the points, with a square for T1‐23 and a triangle for T5‐23. The color of the points indicates the genotype: orange for KWS_199 and blue for SV_207. The height of the bar graph indicates the mean.

Inner, middle and outer leaves of sugar beet were measured for total protein content, protein in extract, protein yield and protein extractability. Leaf samples were collected from two varieties (KWS_199, SV_207) across two locations (Swifterbant (NL) and Wouwse Plantage (NL)). Inner leaves had a higher total protein content (31.6 g kg^−1^) than middle (28.3 g kg^−1^) and outer (21.8 g kg^−1^) leaves (Fig. 1). Samples from trial T1‐23 contained a higher total protein content across all leaf categories, and yielded more protein than trial T5‐23 (Fig. 1(A),(C)). The protein content of the leaf juice extract was highest in middle leaves at 2.2 g kg^−1^ (Fig. 1(B)). This category also showed less variation between plots, in comparison with inner and outer leaves. Middle leaves also yielded the most protein, as a percentage of leaf dry matter (Fig. 1(C)). The extractability of protein increased from inner towards outer leaves. This pattern was observed across both of the trials (Fig. 1(D)).

#### Large differences in variability between plots

By repeatedly sampling a plot, the variability in leaf protein‐related traits among plants within the same plot can be estimated. This variability can be expressed as the CV, with higher values indicating greater variability. The measurement of total protein content, dry matter content and juice percentage had a mean CV across all plots of 3.54, 3.26 and 5.83, respectively. The measurement of the protein in extract had a much higher mean CV of 10.13. For protein yield and protein extractability, which are derivations of the earlier mentioned measurements, the mean CV increased to 12.51 and 12.05, respectively. At times, large variation between the plots was found. For example, the CV of protein extractability in plot 818929 was 6.08, while in plot 819152 it was 15.66, even though this was the same genotype, grown on the same field. Interestingly, the total protein content was higher in samples from trial T1‐23, yet these samples also had a lower standard deviation, resulting in a much lower CV (Table 2). A full table of summary statistics per trait and for each plot is provided in the supporting information (appendix S2).

**Table 2:** CV observed per plot for each trait

Trial	T5‐23				T1‐23				Mean CV across all plots
Genotype	SV_204		KWS_198		SV_204		KWS_198		
Plot number	661009	661046	661035	661049	818733	819206	818929	819152	
*N*	6	6	9	8	8	7	8	9	
*Trait*									
Dry matter	2.85	2.74	4.44	3.58	3.69	2.53	3.03	3.24	**3.26**
Juice	5.09	5.10	5.21	1.58	8.31	7.80	4.57	8.93	**5.83**
Total protein	6.28	4.49	5.60	4.70	1.79	1.75	2.26	1.42	**3.54**
Protein in extract	9.08	17.07	11.92	9.49	6.11	6.88	11.15	9.33	**10.13**
Protein yield	12.61	18.14	15.46	11.89	9.61	10.17	7.05	15.18	**12.51**
Protein extractability	11.24	16.63	16.48	10.08	10.19	10.03	6.08	15.66	**12.05**

**Figure 2:**
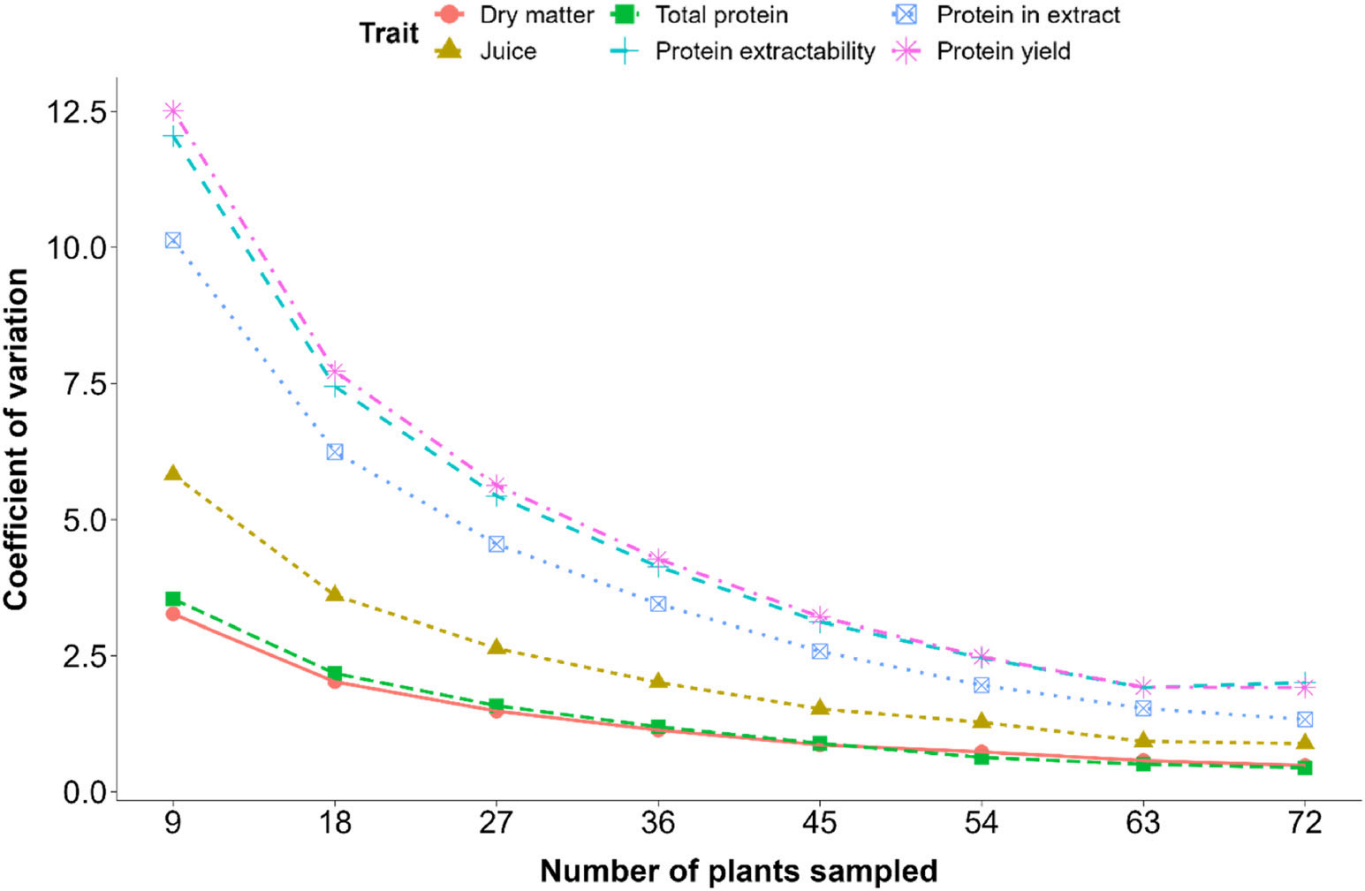
Decrease in the CV for leaf protein‐related traits, by simulating an increase in number of plants sampled. Dry matter content in red, juice percentage in brown, total protein in green, protein extractability in cyan, protein in extract in blue and protein yield in pink.

The measurements on samples of 9 plants were used to extrapolate CVs at larger sample sizes. With increasing sample size, the CV of all parameters decreased (Fig. 2). This decrease was largest when sampling increased from 9 to 18 plants, and second largest from 18 to 27 plants. From 54 plants onwards, the reduction became negligible. The CV was already under 5% at the starting sample size of 9 plants for both total protein and dry matter content. This threshold of 5% was reached at 18 plants for juice percentage, while for the percentage of protein in the extract the sample size had to be increased to 27 plants. For the remaining parameters, protein yield and protein extractability, the sample size had to be increased to 36 plants.

### Is total nitrogen a good measure for protein?

#### Total nitrogen is a good estimate of total protein content

**Figure 3:**
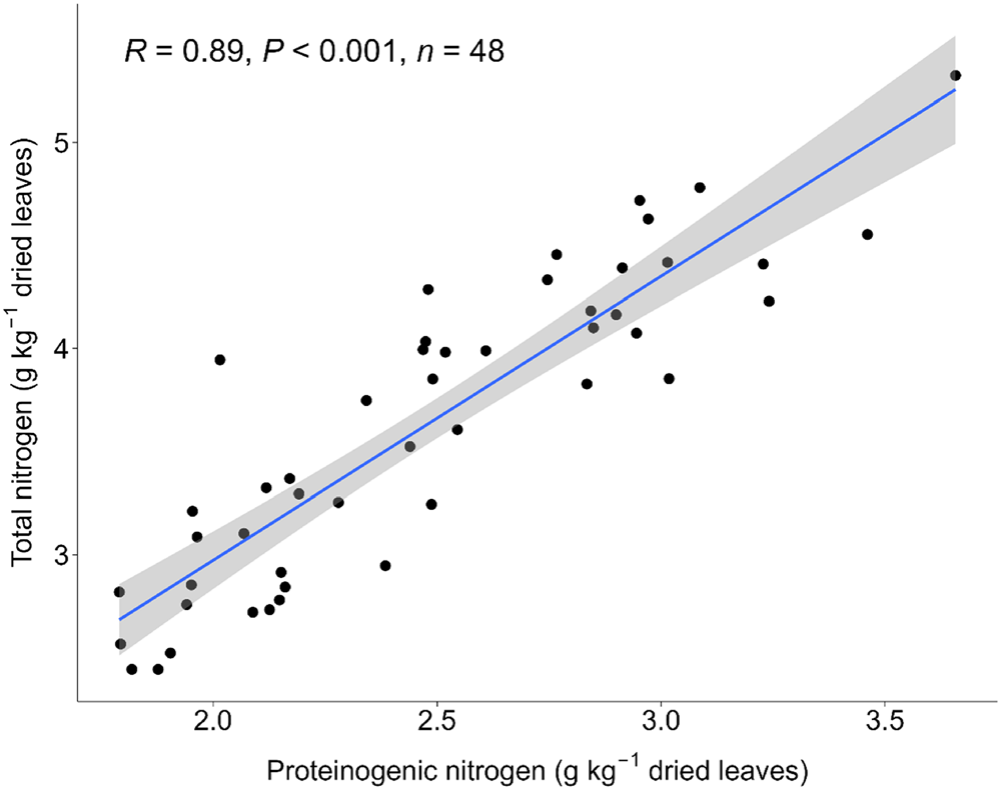
Significant correlation (*R* = 0.89, *P* < 0.001) between total nitrogen and proteinogenic nitrogen, *n* = 48.

Total nitrogen and proteinogenic nitrogen were determined on a subset of 48 leaf samples from the 2022 trials. Total nitrogen and proteinogenic nitrogen are significantly correlated (*R* = 0.89, *P* < 0.001), as measured in dried and milled leaves (Fig. 3). On average, 69% (*σ* = 6%) of the nitrogen in sugar beet leaves derived from proteinogenic compounds. The lowest percentage of proteinogenic nitrogen out of total nitrogen was 51%, while the highest was 80.9%. Using the average of 69%, the actual nitrogen‐to‐protein conversion factor for sugar beet leaves was 4.3, instead of 6.25.

#### Protein content of juice extract is highly correlated with RuBisCO

**Figure 4:**
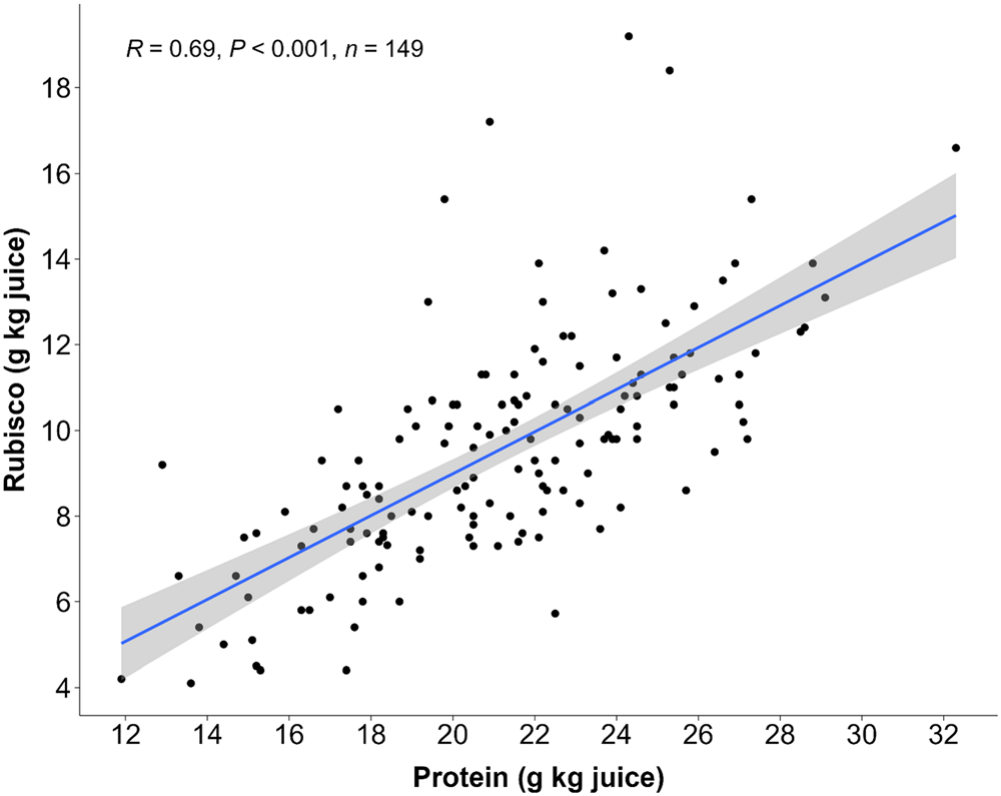
Scatter plot of RuBisCO (g kg^−1^ juice), as measured by size‐exclusion chromatography, *versus* protein (g kg^−1^ juice), as measured by Dumas nitrogen × 6.25.

The measurement of extracted crude protein was found to be significantly correlated (*R* = 0.69, *P* < 0.001) to the measurement of RuBisCO determined by size‐exclusion chromatography (Fig. 4). On average, 44% (*σ* = 9%) of the protein in the centrifuged juice was RuBisCO. While these two measurement methods are strongly correlated, individual samples showed large deviations. The sample with the lowest percentage of RuBisCO out of crude protein had only 25%, while the highest had 82%.

#### Near‐infrared spectroscopy

**Figure 5:**
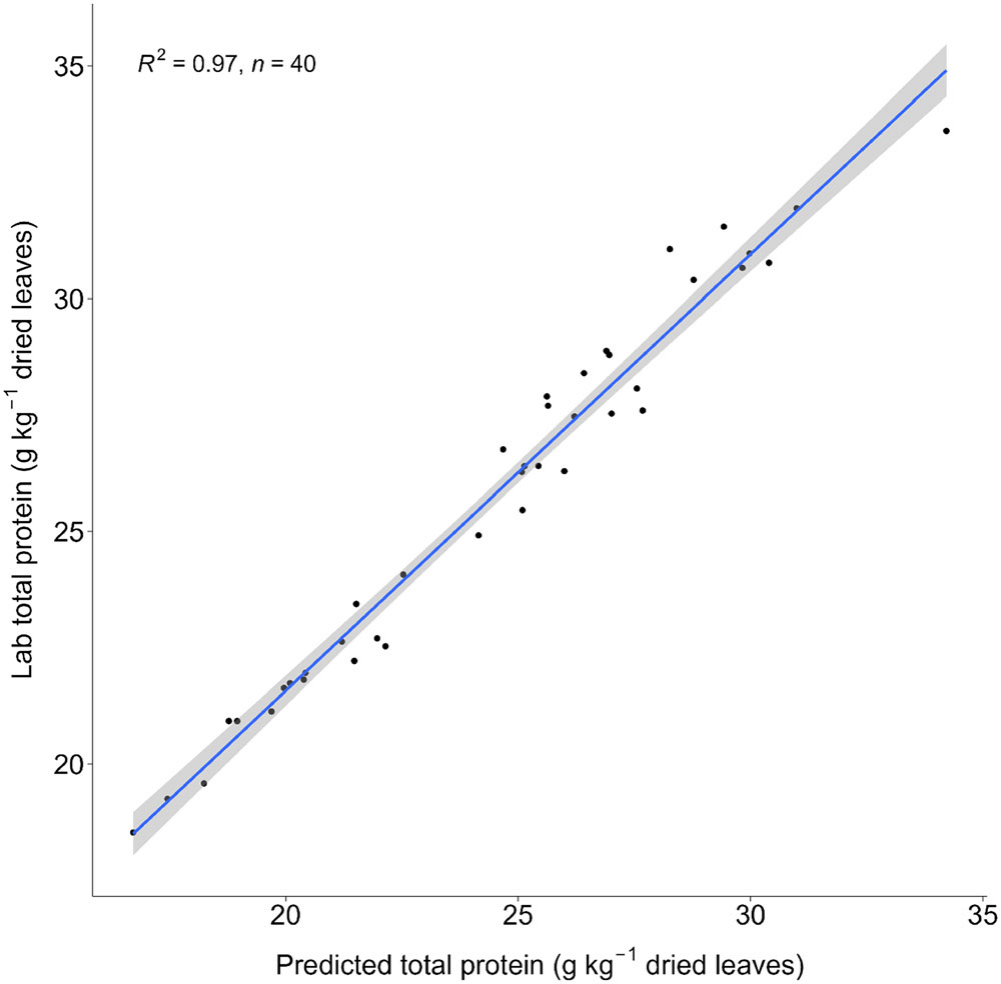
Relation between NIRs‐predicted and laboratory‐measured total protein content of dried sugar beet leaves.

The suitability of NIRs as a phenotyping tool for measuring leaf protein content and extractability was investigated. The total protein content of dried and milled sugar beet leaves is highly predictable using NIRs. External validation resulted in an *R* ^2^ of 0.97 and an RPD of 5.5 (Table 3, Fig. 5). With a reasonably good accuracy, protein yield and RuBisCO yield are also predictable with NIRs with *R* ^2^ of 0.68 and 0.64, respectively. However, when expressing these yields as a percentage of total protein to get protein and RuBisCO extractability, *R* ^2^ decreases to 0 and 0.24, respectively. The protein and RuBisCO content of juice samples can also be predicted from NIR spectra collected directly on the juice. Here, external validation resulted in an *R* ^2^ of 0.6 for protein and 0.72 for RuBisCO (Table 3).

**Table 3:** External validation statistics for NIRs models predicting leaf protein extraction. *R* ^2^, coefficient of determination; SEP(c), standard error of prediction, corrected for bias; RPD, ratio of performance deviation

External validation	Unit	*N*	Biochemical data			NIRs predicted data			*R* ^2^	SEP(c)	RPD
			Mean	Min.	Max.	Mean	Min.	Max.			
*Dried and milled leaves*											
Total protein	g kg^−1^ DM	40	25.77	18.53	33.61	24.48	16.72	34.22	0.97	0.72	5.5
Protein yield	g kg^−1^ DM	29	12.18	7.7	20.42	11.71	8.8	14.3	0.68	1.72	1.66
RuBisCO yield	g kg^−1^ DM	29	4.45	1.67	8.91	4.17	1.81	5.95	0.64	0.94	1.67
Protein extractability	g kg^−1^ protein	29	50.6	37.78	69.53	49.4	47.49	53.2	0.00	6.32	0.98
RuBisCO extractability	g kg^−1^ protein	29	18.19	10	30.1	16.78	12.47	19.95	0.24	3.76	1.15
*Juice*											
Protein	g kg^−1^ juice	27	21.49	15.1	28.5	21.55	16.83	26.83	0.6	2.19	1.58
RuBisCO	g kg^−1^ juice	28	7.44	3.3	11.8	7.49	2.69	11.03	0.72	1.22	1.76

## DISCUSSION

The aim of this research was to develop high‐throughput methods and tools to measure leaf protein extractability in the context of large‐scale breeding trials of sugar beet. Three different approaches were taken. First, the optimal method for obtaining a representative leaf sample of a plot was investigated. Second, the suitability of total nitrogen as a measure of leaf protein content was assessed. Lastly, the potential advantages of NIRs as a phenotyping tool for protein extractability were examined.

### Sampling

Leaf protein content varies between different categories of sugar beet leaves. Inner leaves had the highest protein content, and a decreasing trend was observed from inner to middle to outer leaves. An opposite trend was found for protein extractability, as outer leaves had a higher protein extractability than inner leaves, with middle leaves in between. These results are in line with previous research^20^ where leaves from different positions along a tomato stem are compared. In our study, average protein yield was relatively stable when comparing inner and outer leaves, while total protein content was much lower in outer leaves suggesting that this is the main factor causing the increase in protein extractability in outer leaves. Lower protein content in outer leaves can be explained by a difference in relative leaf age between the different categories. Sugar beet has continuous leaf growth, with new leaves growing from the center of the rosette. Outer leaves are therefore older and are already undergoing leaf senescence when proteins are degraded into peptides and free amino acids, and transported to other parts of the plant. Lower protein content in older leaves has also been reported for other crops, for example for leaves from cassava^32^ and rice.^33^ On average, protein yield was higher from middle leaves compared to inner and outer leaves. We hypothesize that this is due to an increase in the content of RuBisCO in middle leaves due to increased photosynthesis rates, caused by higher light interception as the middle leaves stood tall above those of the other categories. Their position relative to those of the other categories made middle leaves the most accessible for manual sampling, and this will extend to machine‐aided mechanical sampling as well. Furthermore, middle leaves were the most abundant category of leaves. Therefore, they are the most important and representative category of leaves to sample when aiming to assess leaf protein content and yield from a particular sugar beet type.

When comparing leaf samples from the same plot, extensive variation was observed in protein yield, protein extractability and percentage of protein in the juice extract. For dry matter content, total protein content and juice percentage, the samples were more stable. Among the four parameters used to calculate protein extractability, the percentage of protein in the extract showed the largest variability between samples from the same plot. This parameter will therefore also have the largest effect on the variability of protein extractability, and it is worthwhile to investigate this parameter specifically. Aside from differences between the plants in a plot, some of this variability may be explained by the activity of polyphenolic oxidases (PPO) which can induce protein–quinone complex formation, leading to reduced protein solubility.^34^, ^35^ In the process of pressing, subsampling and centrifugation, time varied and this time difference may have led to a higher degree of protein–quinone complex formation in some samples. To prevent PPO activity‐induced errors in the extract measurement, it is advised to add an antioxidant such as sodium metabisulfite during the leaf pressing step, which will inhibit PPO activity.^36^

An increase in accuracy was obtained by increasing the number of plants sampled from the same plot. This was most important for accurate measurements of protein in the extract, protein yield and protein extractability where the number of plants to be sampled lies between 27 and 36. For the dry matter and total protein content, as well as the juice percentage, a relatively low CV (*ca* 5%) was already found using the starting sample size of 9 plants. In previous research, the effect of sample size on the variation of root yield, sugar content and several quality parameters of the beet root was investigated.^21^, ^31^ However, these numbers on root sampling cannot be used for the sampling of leaves. To effectively measure protein extractability in the context of sugar beet breeding trials, we recommend focusing sampling on the middle leaves of plants and avoiding the inner and outer leaves. Additionally, samples should be taken from at least 25 plants. In practice, the most straightforward way to sample is then to take 1–3 middle leaves from all of the plants in the middle row of a plot (*ca* 30 plants). As the beets respond strongly to space, it is best to not sample the border plants at the start and end of a plot, especially if no head rows are grown between the plots.

### Evaluating total nitrogen as a measurement of protein

The percentage of proteinogenic nitrogen from total nitrogen ranged between 51% and 81%, with an average of 69%. Considering that the conventional nitrogen‐to‐protein conversion factor of 6.25 is used with the assumption that all nitrogen derives from protein,^37^ the percentage of proteinogenic nitrogen can be used to recalculate conversion factors. On average, 69% proteinogenic nitrogen corresponds to a nitrogen‐to‐protein conversion factor of 4.3. This value is in line with amino acid analyses of sugar beet leaves from older (6–8 months) plants.^22^ It is also comparable to a general leaf protein conversion factor of 4.43, as reported previously.^38^ In the present study, nitrogen conversion factors for sugar beet leaf protein ranged from 3.2 (51% proteinogenic nitrogen) to 5.1 (81% proteinogenic nitrogen), showcasing that there is variation in this factor between leaf samples originating from different genotypes and environments. Overall, total nitrogen and proteinogenic nitrogen are highly correlated (*R* = 0.89, *P* < 0.001), implying that when selecting for total nitrogen one will be selecting for actual protein content as well.

In the leaf juice extract, RuBisCO was found to make up 44% of the crude protein on average. This is slightly lower than, but comparable to, findings in tomato,^39^ which report that RuBisCO constitutes 52% of the total protein content in the supernatant of centrifuged tomato leaf juice. It is also in line with earlier reports of RuBisCO making up half of the soluble leaf protein fraction.^36^ In this study, RuBisCO levels were found to vary widely, from 25% to 82%. This may be due to variation in the ratio of NPN to protein in the original leaf material. As NPN compounds are small and soluble, they will accumulate in the supernatant. This was shown previously,^40^ where nitrate was one of the NPN compounds that was co‐extracted with the juice fractions during the extraction process. Nitrate and other NPN compounds will therefore have an increased distortion of the actual protein content in the supernatant. Despite this, RuBisCO was strongly correlated (*R* = 0.69, *P* < 0.001) with the total protein content of the supernatant. The relative ease of measuring nitrogen, compared to measuring RuBisCO, is therefore a preferred method for quantifying the extracted protein.

### Advantages of NIRs

NIRs can be used to predict the total protein content of sugar beet leaves. This was expected, as nitrogen and crude protein content has been shown to be highly predictable with NIRs in leaves of other crops.^25^, ^26^ NIRs‐based prediction of protein yield and RuBisCO yield also showed predictive accuracy, with *R* ^2^ of 0.68 and 0.64, respectively. Also, when the spectra are collected on juice samples, protein and RubisCO content can be predicted with reasonably good accuracy, *R* ^2^ of 0.6 and 0.72, respectively. In contrast, NIRs prediction models for protein extractability and RuBisCO extractability perform much worse and should not be used. However, NIRs can be applied to predict the total protein content of dried leaves, and even the extracted protein and RuBisCO contents of leaf juice samples. Therefore, using NIRs will save a lot of time when one has to measure a large number of leaf samples for protein extractability. In the current study, the prediction of total protein content was done with NIR spectra collected from dried and milled leaf samples. The workload could potentially be reduced further by collecting the spectra from fresh leaves. In fresh canopy of sugar beet, visible–NIR hyperspectral imaging has already been used to estimate leaf nitrogen content.^41^ For this, the spectra can be collected using an unmanned aerial vehicle (UAV). UAV‐based multi‐spectral imaging has been applied to estimate the leaf nitrogen content in wheat,^42^ as well as the protein content of silage maize.^43^ However, challenges remain, as UAV‐based imaging is subject to extensive interference due to variability in canopy structure, light, wind and rain conditions as well as other background factors.^44^ To develop a robust prediction model, larger calibration sets across multiple environments may be required. Still, UAV‐based leaf protein prediction would mean that the total leaf protein component in the protein extractability calculation no longer has to be subsampled, dried, milled and measured, vastly reducing the workload.

## CONCLUSION

In order to obtain a plot‐representative sample of sugar beet leaves for measuring protein extractability, at least 25 plants should be sampled. The exact sample size that is needed for accurate results depends on the parameter that needs to be determined. A smaller sample size can be used when determining direct parameters like dry matter and total protein content, while more complex parameters that are derived after processing need larger sample sizes. The workload of measuring protein extractability can be reduced by utilizing NIRs to predict the total protein content of the leaves (starting material). Total nitrogen in dried leaves is strongly correlated to proteinogenic nitrogen; therefore total nitrogen measurements can be used to inform selections for leaf protein content. In the juice extract, the amount of nitrogen correlates with RuBisCO, although this is a weaker correlation compared to total nitrogen and proteinogenic nitrogen in dried leaves. The effect of NPN compounds can be considered as relatively small and RuBisCO accounts for almost half of the extracted protein. Given that measuring nitrogen is simpler and faster than measuring RuBisCO, it is advised to use nitrogen measurements to quantify leaf protein yield. The proposed methods in this study can aid the measurement of leaf protein extraction in sugar beet, but potentially also other crops where leaves are a byproduct, such as potato and carrot. This can help to increase the circularity of sugar beet, and other crops, by valorizing the protein from the leaves.

## Supporting information


**Data S1.** Supporting Information.

## Data Availability

The data that support the findings of this study are available on request from the corresponding author. The data are not publicly available due to privacy or ethical restrictions.
