# Supplementary material for: High‐throughput methods for measuring protein extractability in sugar beet ( Beta vulgaris L.) leaves
Source: J Sci Food Agric. 2025 Jul 21;105(15):8412–21. doi: 10.1002/jsfa.70074 (PMC12595394; doi:10.1002/jsfa.70074)
Supplement: Supplementary file 1 — Data S1. Supporting Information. [file JSFA-105-8412-s001.docx]

# Supplementary

## S1: Sample_size_script.R

### Analyze optimal sample size script. Author: Hugo Rijken

rm(list = ls())

library(readxl)
library(ggplot2)
library(gtools)
library(dplyr)

##
## Attaching package: 'dplyr'

## The following objects are masked from 'package:stats':
##
## filter, lag

## The following objects are masked from 'package:base':
##
## intersect, setdiff, setequal, union

library(ggridges)
library(ggpubr)

data <- read_excel("Results_pre-trial2023_B4P_Project.xlsx")

### Sample size only
data <- data[data$Test == "Sample_size",]


plot_numbers <- unique(data$Plot_nr) # how many individual plots?


#### Calculating CV per trait per field
# storage_list <- list()
my_df <- data.frame(matrix(ncol = 7, nrow = 0))
cols <- c("Trait", "Plot_nr", "K", "Mean", "Sd", "CV", "diff_mean")
names(my_df) <- cols
my_df[,colnames(my_df)] <- lapply(my_df[,colnames(my_df)], as.character)
traits <- c("DM", "Juice_percentage", "protein_dm", "protein_sup", "protein_yield_dm", "protein_extractability")
data[,traits] <- lapply(data[,traits], as.numeric)


for (i in plot_numbers){ # loop over each plot
 #print(i)
 data_subset <- data[data$Plot_nr == i,] # subset for only 1 plot at a time
 #print(length(data_subset$Plot_nr))


 for (t in traits){
 mostinformativemean <- mean(data_subset[[t]])

 highest_k <- nrow(data_subset) # highest_k = number of bags harvested from individual plot
 for (k in 1:highest_k) {
 temp_df <- data.frame(matrix(ncol = 7, nrow = 0))
 colnames(temp_df) <- cols
 res <- combinations(nrow(data_subset), k, repeats.allowed = FALSE) # repeats.allowed = FALSE, so (bag1, bag1, bag1) combi not allowed
 #print(res) # all the combinations for nrow(data_subset) choose k
 for (j in 1:nrow(res)) {
 indices <- res[j, ] # assigns row of combination
 #print(indices)
 values <- data_subset[[t]][indices] # picks the the trait values for that combination
 permutation_mean <- mean(values)
 permutation_sd <- sd(values)
 permutation_cv <- permutation_sd / permutation_mean * 100
 diff_mean <- abs(permutation_mean - mostinformativemean)
 stats <- c(t, i, k, permutation_mean, permutation_sd, permutation_cv, diff_mean) # saves the stats from that combination
 temp_df[j,] <- stats # stores the stats in a dataframe

 }
 my_df <- bind_rows(my_df, temp_df) # adds temp_df rows to previously made my_df

 }
 }
}

## S2. Summary statistics per plot – sampling experiment

| **Trait** | **Field** | **T5-23** | | | | **T1-23** | | | | **Mean across all plots** |
| --- | --- | --- | --- | --- | --- | --- | --- | --- | --- | --- |
|  | **Genotype** | **SES_204** | | **KWS_198** | | **SES_204** | | **KWS_198** | |  |
|  | **Plot number** | 661009 | 661046 | 661035 | 661049 | 818733 | 819206 | 818929 | 819152 |  |
|  | **N** | 6 | 6 | 9 | 8 | 8 | 7 | 8 | 9 |  |
| **Dry matter** | Mean | 12.02 | 12.05 | 11.31 | 12.00 | 9.17 | 8.87 | 9.64 | 8.81 | 10.48 |
|  | Min | 11.61 | 11.74 | 10.52 | 11.58 | 8.67 | 8.49 | 9.22 | 8.35 | 10.02 |
|  | Max | 12.40 | 12.67 | 11.92 | 12.67 | 9.59 | 9.19 | 9.98 | 9.12 | 10.94 |
|  | Std.d | 0.34 | 0.33 | 0.50 | 0.43 | 0.34 | 0.22 | 0.29 | 0.29 | 0.34 |
|  | CV | 2.85 | 2.74 | 4.44 | 3.58 | 3.69 | 2.53 | 3.03 | 3.24 | **3.26** |
| **Juice** | Mean | 79.59 | 76.52 | 75.36 | 77.15 | 76.61 | 77.40 | 78.91 | 78.11 | 77.46 |
|  | Min | 74.48 | 70.53 | 66.99 | 75.68 | 66.24 | 65.89 | 74.56 | 63.34 | 69.72 |
|  | Max | 85.28 | 80.79 | 79.38 | 78.58 | 84.43 | 82.51 | 82.95 | 83.62 | 82.19 |
|  | Std.d | 4.05 | 3.90 | 3.93 | 1.22 | 6.37 | 6.04 | 3.61 | 6.98 | 4.51 |
|  | CV | 5.09 | 5.10 | 5.21 | 1.58 | 8.31 | 7.80 | 4.57 | 8.93 | **5.83** |
| **Total protein** | Mean | 25.08 | 23.84 | 24.73 | 26.31 | 29.01 | 29.47 | 29.10 | 30.48 | 27.25 |
|  | Min | 23.13 | 21.75 | 22.74 | 23.92 | 28.20 | 28.62 | 28.07 | 29.91 | 25.79 |
|  | Max | 27.65 | 24.59 | 26.51 | 28.12 | 29.80 | 30.08 | 30.36 | 31.14 | 28.53 |
|  | Std.d | 1.57 | 1.07 | 1.39 | 1.24 | 0.52 | 0.52 | 0.66 | 0.43 | 0.92 |
|  | CV | 6.28 | 4.49 | 5.60 | 4.70 | 1.79 | 1.75 | 2.26 | 1.42 | **3.54** |
| **Protein in extract** | Mean | 1.92 | 1.82 | 1.88 | 2.01 | 1.84 | 1.80 | 1.89 | 1.73 | 1.86 |
|  | Min | 1.75 | 1.49 | 1.50 | 1.77 | 1.71 | 1.69 | 1.61 | 1.42 | 1.62 |
|  | Max | 2.22 | 2.18 | 2.26 | 2.30 | 1.99 | 1.99 | 2.28 | 1.94 | 2.15 |
|  | Std.d | 0.17 | 0.31 | 0.22 | 0.19 | 0.11 | 0.12 | 0.21 | 0.16 | 0.19 |
|  | CV | 9.08 | 17.07 | 11.92 | 9.49 | 6.11 | 6.88 | 11.15 | 9.33 | **10.13** |
| **Protein yield** | Mean | 12.76 | 11.58 | 12.57 | 12.99 | 15.38 | 15.75 | 15.38 | 15.41 | 13.98 |
|  | Min | 11.04 | 8.86 | 9.62 | 10.95 | 12.82 | 14.31 | 14.25 | 10.61 | 11.56 |
|  | Max | 15.62 | 13.72 | 16.32 | 15.50 | 16.90 | 19.09 | 17.16 | 19.44 | 16.72 |
|  | Std.d | 1.61 | 2.10 | 1.94 | 1.54 | 1.48 | 1.60 | 1.08 | 2.34 | 1.71 |
|  | CV | 12.61 | 18.14 | 15.46 | 11.89 | 9.61 | 10.17 | 7.05 | 15.18 | **12.51** |
| **Protein extractability** | Mean | 50.88 | 48.52 | 51.02 | 49.32 | 53.04 | 53.42 | 52.81 | 50.56 | 51.20 |
|  | Min | 45.54 | 36.37 | 36.65 | 42.21 | 43.26 | 49.02 | 48.95 | 35.37 | 42.17 |
|  | Max | 60.40 | 56.40 | 66.37 | 57.06 | 58.61 | 64.93 | 58.99 | 64.98 | 60.97 |
|  | Std.d | 5.72 | 8.07 | 8.41 | 4.97 | 5.40 | 5.36 | 3.21 | 7.92 | 6.13 |
|  | CV | 11.24 | 16.63 | 16.48 | 10.08 | 10.19 | 10.03 | 6.08 | 15.66 | **12.05** |
